# Ethnoveterinary study of medicinal plants in Malakand Valley, District Dir (Lower), Khyber Pakhtunkhwa, Pakistan

**DOI:** 10.1186/2046-0481-67-6

**Published:** 2014-03-01

**Authors:** Habib ul Hassan, Waheed Murad, Akash Tariq, Ashfaq Ahmad

**Affiliations:** 1 Dir Learning Academy Chaman Abad near GPGC Timergara, Dir (L) 18300, Pakistan; 2 Department of Botany, Kohat University of Science and Technology, Kohat 26000, Pakistan; 3 Department of Botany, Islamia College University, Peshawar 25000, Pakistan

**Keywords:** Ethnoveterinary, Malakand valley, Medicinal plants, Traditional knowledge, Livestock

## Abstract

**Background:**

The Malakand valley of District Dir (Lower) is endowed with a diverse plant wealth. Ethnoveterinary knowledge provides the traditional medicines of livestock that are cheaper than standard treatment and are easily accessible.

**Methods:**

The present study was conducted to document the traditional knowledge of ethnoveterinary practices in Malakand valley, District Dir (Lower). Data was collected from February 2012 to January 2013 by interviewing 120 inhabitants through semi-structured questionnaires. Different questions were asked to collect appropriate data regarding the use of plants for livestock treatment. Direct matrix ranking (DMR) was carried out to find out the use diversity of medicinal plants.

**Findings:**

A total of 28 plants belonging to 23 families were collected and identified for the treatment of livestock in the study area. Majority of the plants were collected from wild (68%) habitat and very few from cultivated sources. The leaves (28%) were identified as the major plant part for herbal preparations. The most frequent ailments of livestocks in the study area were stomach disorders and *Charmaikh* (local disease name). Various ingredients were used along with ethnoveterinary medicines i.e. sugar, flour, milk etc. The elder people of the village had a rich knowledge as compared to the young generation. According to DMR output, *Monotheca buxifolia* ranked first and *Dalbergia sisso* and *Melia azedarach* ranked second due to high multipurpose uses among all species and was found most threatened in the study area.

**Conclusion:**

It has been concluded that the native of the region heavily dependent on medicinal plants for the treatment of variety of livestock ailments. Traditional knowledge always provides a baseline for further phytochemical and pharmacological investigation. The documentation of the ethnoveterinary practices in study area was necessary before this precious knowledge is lost forever due to rapid socioeconomic, environmental and technological changes.

## Background

Plants are being used for medicinal purposes by local inhabitants since earliest times. It is a recognized fact that plants serve a potent medicine for curing various diseases [1]. Ethnoveterinary medicine generally means the folks beliefs, knowledge skills, methods and practices pertaining to the health of animals, which play a vital role in rural areas as chief source of medicine being used to cure livestock [2]. The use of ethnoveterinary medicine presents a cheaper and sustainable alternative to synthetic medicines [3]. Ethnoveterinary medicines are as older as the domestication of various animal species. These herbal preparations, drawing upon centuries of traditional belief and use, are in practice over time by pastoralists and farmers for the treatment of different diseases of livestock [4].

Pakistan is an agricultural country and upto 80% of the population is dependent on agriculture and livestock [2,5]. The significance of livestock can be guessed from the fact that Pakistan is the fifth largest milk producing country of the world. Majority of Pakistani livestock farmers are poor and own 5–6 animals per family [5]. Most of these farmers cannot afford modern allopathic drugs due to financial constrains, which ultimately leads to poor livestock production and monetary losses due to poor health of animals. Under such conditions, ethnoveterinary medicine can be promoted as an alternative of modern drugs and it will help in poverty alleviation by empowering the people to make use of their own resources for healing of their livestock. Despite the advancement of pharmaceutical industry and development of clinical agents, traditional indigenous medicine is still practiced in rural areas for human and livestock ailments. Draw backs to modern veterinary practice include questionable quality of allopathic drugs, development of chemo-resistance in livestock and user unfriendly effects such as high antibiotic and hormone residues in the milk and other animal products [6]. Ethnoveterinary medicine practice is used for the maintenance of good animal health in developing countries [7]. Livestock farmers all over the Pakistan can draw on over 4000 years of knowledge and experience. Traditional veterinary medicine knowledge like all other traditional knowledge systems is handed down orally from generation to generation and it may disappear because of rapid socioeconomic, environmental and technological changes and as a result of the loss of cultural heritage under the guise of civilization [8]. In order to conserve the traditional knowledge, the documentation through systematic studies is imperative before it is lost forever.

Tremendous work has been done world widely on the documentation of ethnoveterinary practices. Pal [9], McCorkle [10], Pande and Kumar [11], Catley and Mohammad [12], Kohler-Rollefson and Rathore [13], Lans *et al.*[14], Heffernan *et al.*[15], Wanyama [16], Waihenya *et al.*[17,18] Tabuti *et al.*[19] and Yirga *et al.*[20] have documented the medicinal plants used to cure animal diseases, but in Pakistan very little attention has been given on the use and documentation of plants as veterinary medicines and a dire need is raised to record this knowledge. Some workers have documented the indigenous ethnoveterinary practices in different parts of Pakistan [21-27] but documentation of such study in research area was not done before, therefore, it is necessary to conserve the ethnoveternary indigenous knowledge of Malakand valley, Dir (L). This is the first attempt to elucidate the ethnomedicinal uses of plants as veterinary medicines in the study area and it is imperative to know the ethnoveterinary medicinal plants of unexplored region like Malakand valley of District Dir (L), Pakistan. Consequently, the present study was designed with the aim to document the reliable information on indigenous ethnoveterinary knowledge of traditional healers and to provide baseline information for further chemical and pharmacological investigation for the advancement and improvement in animal drugs system.

## Methods

### Study area

The present study was conducted in District Dir (L) Khybr Pakhtunkhwa (KP), Northern Pakistan. It lies in Hindu Kush range between 35° 10 to 35° 16 N Latitude and 71° 50 to 71° 83 E Longitudes [28]. Dir (L) has an area of 1585 km^2^ and having the population of 717649 with a density of 543.3 people per kilometer [29]. Most of the people live in the rural hilly areas and still depend on natural forest resources and traditional methodologies to cure different ailments of animals. Documentation was done in the region of Malakand valley. It was found that the valley has retained rich diversified flora. The valley elevation ranges from 1200 m to 2800 m above sea level [28]. In most parts of the valley, the local populace rear cattle for their own aims execution like bull for plough, cows, buffaloes, sheep and goats give milk, butter, curd and meat. The inhabitants of this area use several plants for the treatment of various diseases in animals.

### Compilation of data

Information regarding ethnomedicinal plants pertaining to livestock ailments in Dir (L) was collected by interviewing 120 local inhabitants during four different visits conducted from February 2012 to January 2013. For this purpose a semi-structured questionnaires was prepared. During interview data regarding the type of livestock ailment treated or prevented and the types of medicinal plants used pertaining their local names, plant parts used, the mode of remedy preparation, single or mixture of plants used and ingredients utilized along with remedies. Data was collected by interviewing different ethnic groups (Local villagers, *afghan refugees* and *gujjars*) of the region. In the field wok preference was given to old age villagers (n = 80) due to the possession of sound knowledge regarding the uses of ethnoveterinary medicinal plants and *Afghan refugees* (n = 20) and the *Gujjars* (n = 20) of the area. Data on use diversity of multipurpose medicinal plants were evaluated by a direct matrix ranking (DMR) exercises as described in Cotton [30] that involved fifteen (ten men and five women) key informants. Participants for this exercise were selected based on their long years of experience as traditional herbal practitioners in the study area as described in Yineger et al. [31].

### Preservation of plants

Plant specimens were collected, dried and mounted on standard herbarium sheets. Scientific names (Latin), family names and publication authors were corrected according to the Flora of Pakistan [32]. Confirmation of plants was done by comparing them with the already identified plant specimens preserved in the herbarium of University of Peshawar, Pakistan. The correctly identified specimens were deposited in the herbarium, Department of Botany, Kohat University of Science & Technology, Kohat.

## Results

**Figure 1:**
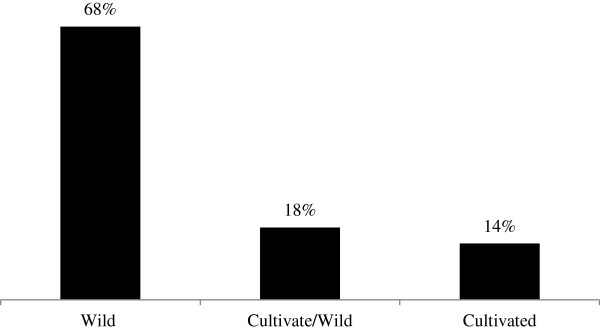
The sources of ethnoveterinary plants.

**Figure 2:**
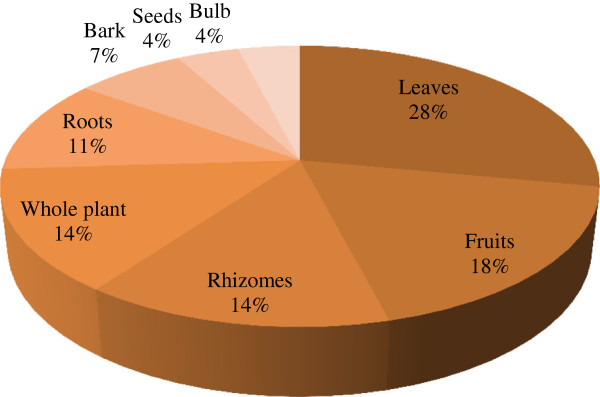
The use of plant parts involved in remedy preparations.

A total of 28 plants belonging to 23 families were documented. Two plants each of family Solanaceae, Alliaceae, Myrtaceae and Lamiaceae were commonly used (Table 1). Moreover, wild plants (68%) were mostly collected for livestock treatment followed by cultivated as well as wild (18%) and cultivated plants (14%) (Figure 1). Mostly the leaves (28%) of the plant were used by the local inhabitants for curing different ailments of livestock (Figure 2). The most common ailments of livestock documented in study area were abdominal pain, *Charmaikh* and flatulence (Table 1). It was observed that out of 120 respondents, 90 were old age (40 to 80 years) people and 30 were young (below 40 years) people of the region. The output of the DMR exercise on ten multipurpose medicinal plants enabled to identify which of the multipurpose plants is most under pressure in the area and the corresponding factors that threaten the plant. Accordingly, *Monotheca buxifolia*, ranked first (most threatened); *Dalbergia sisso* and *Melia azedarach* ranked second; *Poenia emodi* ranked third (Table 2). Results also indicated that those multipurpose medicinal plant species are currently exploited more for fodder, fuel, construction and agricultural tools purposes than for their medicinal role.

**Table 1:** Ethnoveterinary uses of medicinal plants of the study area

**Scientific names**	**Local names**	**Family**	**Part used**	**Ethnoveterinary uses**
*Acorus calamus* L.	Skhawaja	Araceae	Rhizome	The powdered rhizome is used to increase body temperature of cattle and to overcome local disease name charmaikh.
*Allium cepa* L.	Piaz	Alliaceae	Bulb	Bulb is used to relief abdominal pain in all types of cattle.
*Allium sativum* L.	Oga	Alliaceae	Rhizome	The crushed paste of rhizome mixed with flour is used to bring the cows in heat and to become pregnant.
*Ammi visnaga* (L.) Lam	Sperky	Apiaceae	Fruits	The fruits decoction is used for abdominal pain and to increase the body temperature (charmaikh).
*Berberis lycium* Royle	Kwary	Berberidaceae	Roots	The derived extract after crushing and boiling roots is used for the internal injuries and for body warmth in cattle.
*Brassica compestris* L.	Sharshum	Brassicaceae	Seeds (oil)	The oil extracted from the seeds is used to overcome on flatulence.
*Chenopodium album* L.	Sarmy	Chenopodiaceae	Whole plant	Crushed plant body is used for flatulence.
*Citrullus colocynthesis* (L.) Schrad.	Khroendwana	Cucurbitaceae	Fruits	A mixture of crushed fruit powder and flour paste is used for abdominal pain.
*Dalbergia sissoo* Roxb.	Shawa	Papilionaceae	Bark	Crushed bark formula given along with paste of flour as remedy for the abdominal pain.
*Datura stramonium* L.	Bathora	Solanaceae	Leaves	The leaves along with piece of bread are directly used to increase body temperature (charmaikh) in buffaloes.
*Dodonaea viscosa* (L). jacq.	Ghwaraskey	Sapindaceae	Leaves	The crushed leaves powder is scattered on open wound for earlier healing and treatment in all types of cattle.
*Geranium wallichianum* D.Don ex. Sweet	Srazela	Geraniaceae	Rhizome	The rhizome powder is used to promote lactation in cows and buffaloes.
*Grewia optiva* J.R. Drumm.ex.Burret.	Pastawony	Tiliaceae	Bark	The stem bark is boiled in water and the extracted water is used to induce diarrhea so as to overcome on their body weakness and to gain body weight.
*Hypericum perforatum* L.	Shenchay	Hypericaceae	Whole plant	The boiled extract of the plant body is used to increase body temperature of cattle particularly of cows.
*Juglans regia* L.	Ghoz	Juglandaceae	Leaves	The leaves are fed to cows for the expulsion of retention of placenta after parturition in all types of cattle.
*Mallotus philippensis* (Lam.) Moll. Arg.	Kambela	Myrtaceae	Fruits	The crushed fruits are used to increase body temperature (charmaikh).
*Malva neglecta* Wall.	Panerak	Euphorbiaceae	Leaves	Crushed and boiled roots extract is used for the lowering of flatulence.
*Melia azedarach* L.	Shunday	Meliaceae	Leaves	Leaves are rubbed on snake bite region to remove the venom while crushed bark is a useful remedy for flatulence.
*Mentha longifolia* (L.) Huds.	Wenalay	Lamiaceae	Roots	Crushed roots are boiled in water and then given to cattle in case diarrhea and for low body temperature (charmaikh).
*Monotheca buxifolia* (Falc.) A.D.	Gwargora	Sapotaceae	Leaves	The leaves are directly fed to the cows to treat the abnormal taste of milk, after 2 to 4 days the milk retains the original taste.
*Myrtus communis* L.	Mano	Myrtaceae	Leaves	The leaves decoction is used as anti-diarrheal for cattle.
*Nicotiana rustica* L.	Tamako	Solanaceae	Leaves	The extract of leaves is externally rubbed on the body to remove the ticks and ectoparasites found in cattle.
*Paeonia emodi* Wall.ex.Hook.	Mamekh	Paeoniaceae	Roots	Paste prepared from crushed tuber along with maize flour against internal injuries and to strengthen bulls.
*Quercus balooth* Griffith	Zagwana	Fagaceae	Fruits	The fruit are directly fed to the cattle for urinary tract diseases.
*Rheum emodi* Wall.ex.Miessn	Chotyal	Polygonaceae	Whole plant	The decoction of the plant is useful to strengthen the cows and bulls and to triumph over their body weakness.
*Rumex hastatus* D. Don	Trokay	Polygonaceae	Whole plant	The plant body is fed to cattle when they deny eating so to increase their appetite.
*Salvia moorcroftiana* Wall.ex. Benth	Kharghwag	Lamiaceae	Roots	The boiled root extract is used for the internal injuries of cattle.
*Zanthoxylum armatum.* Steud	Dambara	Rutaceae	Fruits	The paste of crushed fruits mixed with flour and gurh is used to induce heat in cow for early pregnancy.

**Table 2:** Average direct matrix ranking (DMR) score of fifteen key informants for ten medicinal plants species

**Use diversity**	***M. azedarach***	***M. buxifolia***	***D. sisso***	***J. regia***	***P. emodi***	***Q. balooth***	***R. hastatus***	**Total**	**Rank**
Fodder	3	4	3	3	4	3	3	23	1
Fuel	4	3	3	2	2	2	2	17	3
Construction	2	3	5	3	2	2	2	19	2
Agriculture	3	4	4	2	2	2	2	19	2
Medicinal	4	4	1	3	5	3	3	23	1
Total	16	18	16	13	15	12	12		
Rank	2	1	2	4	3	5	5		

## Discussion

Farmers and pastoralists in several countries use medicinal plants in maintenance and conservation of the livestock health care. More than 70% of the Pakistan population lives in rural areas and is directly or indirectly linked with agriculture for their livelihood. Livestock, the largest contributor to overall agriculture value added (contributing 49%), grew by 2.6% in 2003–04 as against 2.8% in 2002–03. The role of livestock in rural economy may be realized from the fact that 30–35 million rural populations is engaged in livestock raising, having household holdings of 2–3 cattle/buffalo and 5–6 sheep/goat per family which help them to derive 30-40% of their income from it. The livestock include cattle, buffalos, sheep and goats [33].

The farmers and nomadic people of the area are not only depending on wild plants to get fodder for their animals but also use different medicinal plants to treat various animal diseases. In plant parts the leaves were mostly used for livestock remedy preparation [20,34,35]. A study conducted by Poffenberger *et al.*[36] indicated that collection of leaves poses no significant threat to the survival of individual plants as compared to underground part, stem, bark and whole plant. The use of specific plant parts suggests that these parts have strongest medicinal properties but it needs biochemical analysis and pharmaceutical screening to cross-check the local information.

The method of drug preparation in many cases varied from individual to individual. The same plant material for the same ailment was prepared in different ways by different traditional veterinary healers. In Malakand valley, the plants parts are mainly used for livestock treatment in grind/crushed and boiled form. According to Deeba [37] grinding or crushing and soaking or boiling is the most common method of drugs extraction. Preparations of remedies in study area involved single medicinal plant, which is in agreement with the findings of studies conducted elsewhere in Ethiopia [38,39] while it is believed that the potency of plant remedies could be enhanced when they are used in concoction form [40]. The most frequent ailments in the area was stomach related disorders and to induce heat in the body of livestock during winter season (Local disease *Charmaikh*), 5 plants each are used to treat such types of ailments (Table 1). Generally a single plant is used for a variety of livestock disorders like *Melia azedarach* is used for flatulence and snake bite. Similar studies were also reported by Khan *et al.*[41] and Monteiro *et al.*[42] from Shawar Valley district Swat Pakistan and from Marajó Island, Eastern Amazonia, Brazil where they mentioned multiple uses of plants. It was observed that nearly all plants are used in combination with other ingredients or vehicles like flour, sugar (gurh), milk etc. owing to the astringent taste of the remedies. It has been suggested that the use of such vehicles may dilute or reduce the relative potency of the drug [21]. Most of the plants used for livestock treatment belong to the family Solanaceae, Alliaceae Polygonaceae, Myrtaceae and Lamiaceae. Rashid *et al.*[43] also reported similar studies of plants from Netrakona district Bangladesh. Dominance of medicinal plant species from aforementioned families could be attributed to their wider distribution and abundance in the area. Moreover, the wide utilization of species from these families might relate to the presence of effective bioactive ingredients against livestock ailments [44].

The young generation had little knowledge about the traditional medicines while the elder people know much more about the traditional knowledge to treat livestock ailments. This finding line with the study of Yirga *et al.*[45] reported that majority of the respondents were old age people and very few youths were involved in traditional livestock treatment. During research study, it has been observed that most of the plants are collected from wild to treat different diseases of livestock and this has been creating a pressure on the population of wild plants in addition to environmental degradation and deforestation. The basic reasons behind that most of the plants are easily available near the sites of housesteads and easily accessible as compared to cultivated plants. Same finding was also recorded by Giday and Tilahun [46] that the use of uncultivated plants is more common in Ethiopia as compared to cultivated plants.

The output of a DMR showed highest values (ranks) for a number of multipurpose medicinal plants of the study area such as *Monotheca buxifolia*, *Dalbergia sisso*, *Melia azedarach*, *Poenia emodi*, *Juglan regia*, *Quercus balooth* and *Rumex hastatus*. The result indicates that these plants are exploited more for their non-medicinal uses than for reported medicinal values. Overharvesting of multipurpose medicinal plant species for construction, fuel wood, fodder agricultural tools and other purposes were found the responsible factors aggravating depletion of the species in the area. Thus, the result calls for an urgent complementary conservation action to save the fast eroding multipurpose medicinal plant species of the area. Same pattern of highest exploitation of multipurpose medicinal plants for uses other than their traditional importance has been found [31].

## Conclusion

Ethnoveterinary research has made it possible to find out some active constituents from therapeutic plants, so these motivating ethnomedicinal findings can be research provoking for future. Ethnobotanical knowledge is under severe threat of urbanization, expanding agricultural demands and acculturating trend of village people. Due to urbanization, the ethno-medicinal knowledge can be lost in future, so it is a dire need to collect and systematically document this precious and empirical folklore knowledge and pay due consideration to protect and conserve wild medicinal plants. Ethnoveterinary plants and remedies documented here need phytochemical and pharmacological screening for active principles and clinical trials for therapeutic actions. The use of herbal medicines in the research area could most likely be promoted and strengthened by initiating a coordinated programme of research and development for evaluating and testing the efficacy of the plants in use by standardizing methods for cultivation and preservation of plants.

## Competing interests

The authors declare that they have no competing interests.

## Authors’ contributions

HUH conducted the field work, AT has analyzed the data. AT and AA have written the whole draft of this report. WM supervised all stages of the work presented in this report and provide comments on the report. All authors read and approved the final manuscript.
